# Distinct Features of Doublecortin as a Marker of Neuronal Migration and Its Implications in Cancer Cell Mobility

**DOI:** 10.3389/fnmol.2017.00199

**Published:** 2017-06-28

**Authors:** Abiola A. Ayanlaja, Ye Xiong, Yue Gao, GuangQuan Ji, Chuanxi Tang, Zamzam Abdikani Abdullah, DianShuai Gao

**Affiliations:** Xuzhou Key Laboratory of Neurobiology, Department of Neurobiology and Anatomy, Xuzhou Medical UniversityXuzhou, China

**Keywords:** DCX-doublecortin, MT-microtubule, NSC-neural stem cells, CSC-cancer stem cells, GBM-glioblastoma multiforme, MAP-microtubule-associated protein

## Abstract

Neuronal migration is a critical process in the development of the nervous system. Defects in the migration of the neurons are associated with diseases like lissencephaly, subcortical band heterotopia (SBH), and pachygyria. Doublecortin (DCX) is an essential factor in neurogenesis and mutations in this protein impairs neuronal migration leading to several pathological conditions. Although, DCX is capable of modulating and stabilizing microtubules (MTs) to ensure effective migration, the mechanisms involved in executing these functions remain poorly understood. Meanwhile, there are existing gaps regarding the processes that underlie tumor initiation and progression into cancer as well as the ability to migrate and invade normal cells. Several studies suggest that DCX is involved in cancer metastasis. Unstable interactions between DCX and MTs destabilizes cytoskeletal organization leading to disorganized movements of cells, a process which may be implicated in the uncontrolled migration of cancer cells. However, the underlying mechanism is complex and require further clarification. Therefore, exploring the importance and features known up to date about this molecule will broaden our understanding and shed light on potential therapeutic approaches for the associated neurological diseases. This review summarizes current knowledge about DCX, its features, functions, and relationships with other proteins. We also present an overview of its role in cancer cells and highlight the importance of studying its gene mutations.

## Introduction

Microtubules (MTs) are essential for a variety of cellular functions, notably, cell motility, transport, polarity and shape, as well as mitosis. Amongst these cellular processes, is the ability of MTs to function properly in their intrinsic dynamic state. However, these functions can only be possible when MTs are regulated by molecules known as microtubule-associated proteins (MAPs). MAPs can either influence stabilization and destabilization of MTs, orchestrate cellular dynamics, or serve as a linker between cytoskeletal components (Maiato et al., 2004). Doublecortin (DCX) is a unique MAP that exhibits these features collectively. Interestingly, DCX may also be implicated in the development of cancer cells due to its significance in the migration of neuroblasts. In this review, we will summarize current features of the neuronal MAP DCX, its expression in neurons and other tissues of various species, its roles in neurological disorders as well as its importance in cell proliferation during neurogenesis. We will discuss the mechanisms underlying MT stabilization via DCX regulations and the links between MTs and filamentous actin. We will also describe the implications of MT stabilization by phosphoregulation of DCX in cancer cell migration and possible problems that define the field. Finally, we will discuss the similarities between DCX superfamily members and the importance of studying DCX.

### DCX is essential for the development of a functional brain

DCX is a 40 kDa phosphoprotein encoded by the *DCX* gene. It is a nervous system-specific MAP expressed in migrating neurons of the central and peripheral nervous system during embryonic and postnatal development (Gleeson et al., 1999). It was first described in 1998 (Gleeson et al., 1998) with regards to its mutation in human X-linked lissencephaly and double cortex syndrome (known as SBH); two neurodevelopmental disorders associated with the abnormal migration of cerebral cortical neurons (Sossey-Alaoui et al., 1998; Feng and Walsh, 2001). Lissencephaly and SBH patients display a range of symptoms including epilepsy, intellectual impairment, and infant death which result from abnormal development of the cerebral cortex. Mutations of the DCX protein causes defective neuronal migration in a way that the properly structured layers of the cortex are poorly organized. Because the DCX gene is on the X chromosome, females with a DCX mutated genotype exhibit random inactivation in one of the two X chromosomes; this ensures that half of the cells have a functional copy of the gene and migrate correctly into a layered cortex. In contrast, the other half does not have a functional copy and subsequently stop half-way along their journey through the developing cortex. This creates heterotopic bands of gray matter in between the cortex and the ventricle, thus the so-called double cortex. On the other hand, hemizygous mutations occur in male patients who possess no functional copy of the DCX protein resulting in lissencephaly. Their cortex is abnormally thick and composed of four poorly organized layers, hence they exhibit more severe symptoms (Gleeson, 2000).

Kim et al. recently reported a novel missense mutation of DCX linked to late childhood-onset familial focal epilepsy and anterior dominant pachygyria without SBH in both male and female (Kim et al., 2016). In this case, transient focal seizures occurred in patients accompanied with tonic or dystonic activities in an age-dependent manner. Similarly, like lissencephaly patients, hemizygous males displayed more frequent seizures and abnormal development coupled with generalized pachygyria. On the flip side, heterozygous female patients exhibited only anterior pachygyria. This novel DCX mutation is found in the N-terminal region of the N-DC domain (Kim et al., 2016).

In addition, DCX has also been reported to be crucial in the proliferation of progenitor cells during neurogenesis in DCX mutant embryonic brains. Abnormal neuronal migration combined with defective proliferation was reported in DCX mutant mouse, although cortical organization was fairly disrupted. Pramparo et al. reported irregularities in spindle orientation of radial glial progenitors in DCX/LIS double knockout neurons compared with wild-type neurons. This correlates with differences in the number of bromodeoxyuridine (BrdU)-positive cells, as cell circle enters quiescent stage frequently when compared with the wild-type neurons (Pramparo et al., 2010). In the absence of DCX, significant depletion of the progenitor pool during cortical development was observed. These findings imply that the functions of DCX is not limited to neuronal migration but is also crucial for cellular proliferation during neurogenesis.

### A principal mediator influencing the activities of neuronal migration and MT stabilization

**Figure 1:**
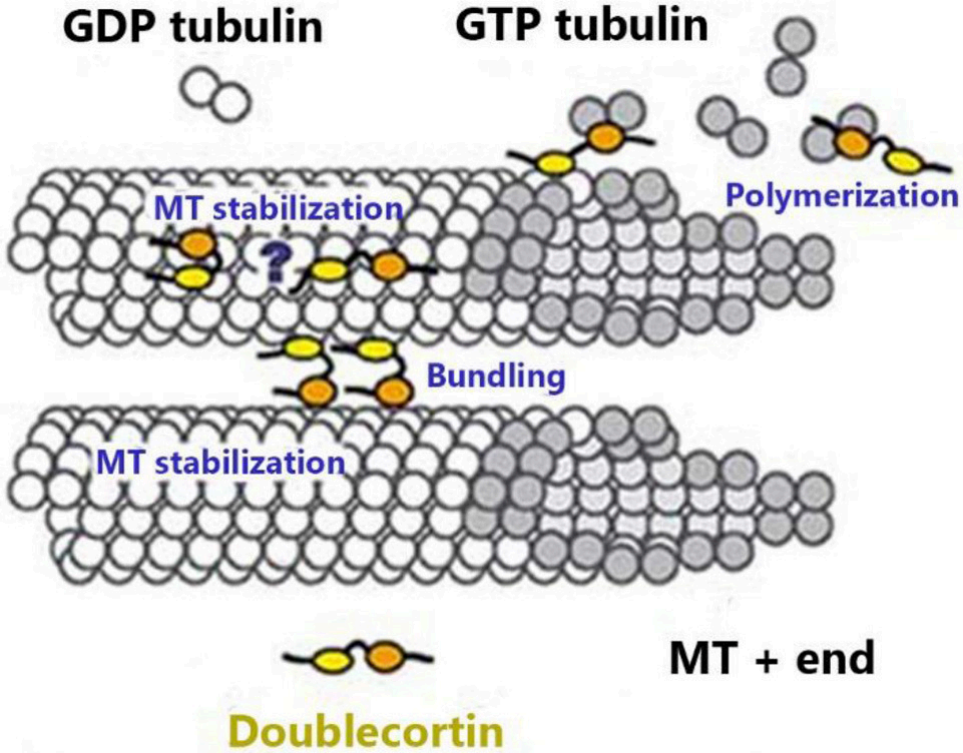
MTs are hollow tubes made of pfs, each of which is made of α and β-tubulin monomers tightly bound together in an organized conformation. Both monomers are composed of GTP molecules. This fig. shows DCX binding to the tubulin monomers at the MT plus-end of the β-tubulin (which possesses the hydrolyzed GDP) fitting into the inter-pf valley. DCX stabilizes MT, promotes bundling, and favors MT polymerization (Moores et al., 2003).

DCX is essential for neuronal differentiation and migration of human neurons by virtue of its involvement in MT stabilization. It is highly expressed at the axon of neurons and may regulate MTs in response to extracellular signals in these distal zones to facilitate pathfinding during development (Gleeson et al., 1999; Weimer and Anton, 2006; Tint et al., 2009). DCX is a determinant factor in growth cone formation, dendritic extension, nuclear translocation, and it also prevents nucleokinesis defects (Burgess and Reiner, 2000; Friocourt et al., 2003; Moores et al., 2006; Koizumi et al., 2006a). DCX has been shown to influence the structure of MTs as it facilitates the binding of protofilaments (pfs) and inhibits depolymerization of MTs (Figure 1); this is regarded as one of its most important features because of its distinctive role in nucleating and binding MTs with a 13-pf construction (Moores et al., 2004; Bechstedt and Brouhard, 2012). This function is particularly important because MTs polymerized with DCX presented 13 pfs whereas those polymerized in the absence of DCX varied from 8 to 19 (Matsuo et al., 1998; Brown et al., 2003; Couillard-Despres et al., 2005).

### Expression of DCX is generalized in many species

Studies have shown the expression of DCX in various regions of the developing nervous system. It is highly expressed in newly produced cells in the neurogenic zones; the subventricular zone (SVZ) along the lateral ventricle and the subgranular zone (SGZ) of the dentate gyrus. In the neurospheres derived from SVZ of adult rodents, it was demonstrated that DCX promoted cell migration during neurogenesis (Ocbina et al., 2006; Jin K. et al., 2010) due to its expression around the focal cortical infarcts in migrating neuroblasts during a short phase of their growth, in both adult and developing mammals (Matsuo et al., 1998; Gleeson et al., 1999). The DCX-positive cells move along the rostral migrative stream to the olfactory bulb of the adult rodent brain (Gleeson et al., 1999; Couillard-Despres et al., 2005; von Bohlen und Halbach, 2011). Pechnick et al. further illustrated that 2.6% of hippocampal cells of wild-type mice were DCX-positive. Interestingly, in these cells, DCX co-localized with BrdU in the SGZ (Pechnick et al., 2008). This implies that DCX-positive cells are required to maintain the progenitor pool and that enhanced neurogenesis is responsible for increased cellular proliferation.

Indeed, DCX has been adopted as a marker for neuronal precursors and migrating neuroblasts during adult neurogenesis (Brown et al., 2003; Couillard-Despres et al., 2005; von Bohlen und Halbach, 2011; Wang C. et al., 2011). It plays a crucial role in pathologic conditions as it is required in maintaining the progenitor pool (Couillard-Despres et al., 2006; Zhao et al., 2008; Marin et al., 2010; Pramparo et al., 2010) by providing newly generated cells migrating out of the point of origin toward the lesion or an injured area to replace neurons (Liu et al., 2008). DCX is expressed in the brain of several species and has consequently been studied for its involvement in neurogenesis, cellular movement, wound healing, and neuronal plasticity. It is expressed in the retinas of chick, rat, shark, and most recently the sea lamprey (Wakabayashi et al., 2008; Kim and Sun, 2012; Sanchez-Farias and Candal, 2015; Fernandez-Lopez et al., 2016). It is also expressed in short-lived annual fish (teleost *Nothobranchius furzeri*) (Tozzini et al., 2012), the zebra finch (*Taeniopygia guttata*; Itoh et al., 2006), in rat retinal pigment epithelium (RPE) cells *in vitro* (Engelhardt et al., 2005), as well as in immature and adult neurons in the cerebellum of guinea pig, cat, dog, and primates (Couillard-Despres et al., 2005; Ocbina et al., 2006; De Nevi et al., 2013). Moreover, DCX has been explored for its role in brain plasticity by studies in seasonal songbirds such as canaries (Balthazart et al., 2008; Yamamura et al., 2011), chickadees (*Poecile gambeli*; Fox et al., 2010), sparrows (*Zonotrichia leucophrys*; LaDage et al., 2010), quails (*Coturnix japonica*; Hall and Macdougall-Shackleton, 2012), and starlings (*Sturnus vulgaris*; Migaud et al., 2010). Hence, DCX is being utilized as a marker for detecting the processes of neural recruitment. Consequently, adult-born neurons are detected in the hypothalamus of several mammals including sheep, mouse, rats, vole, and hamster (Fowler et al., 2005; Xu et al., 2005; Pierce and Xu, 2010; Migaud et al., 2011; Mohr and Sisk, 2013; Robins et al., 2013; Batailler et al., 2014), suggesting that hypothalamic neurogenesis is a conserved process in mammals. Meanwhile, microchiropterans were previously reported to undergo no adult hippocampal neurogenesis (Amrein et al., 2007), later studies reported otherwise with significant expression of DCX detected via immunohistochemistry (Chawana et al., 2014).

In addition, despite DCX specificity in central nervous system (CNS), a recent study has confirmed the expression of DCX in the cytoplasm of beta cells and has been postulated to be a possible biomarker for beta cell injury (Jiang et al., 2013). This may be due to the supply of numerous nerves from the autonomic nervous system to the endocrine pancreas (Begg and Woods, 2013). This further exposes the versatility of the DCX protein in cellular activities and can open a broad spectrum as to the relationship between neurogenesis and the production of new beta cells in response to diabetes. These links require further clarification, and its functions in these cells should be explored.

Collectively, these findings indicate that DCX is expressed in different species during neurogenesis and it may be utilized as a marker for neuronal migration as well as an indicator of neurogenesis. Owing to the limitations in other methods, DCX can be considered a more accurate indicator of newly generated cells in the CNS. For instance, retroviral incorporation in animal models may cause lesions in the parenchyma, resulting in inflammatory reactions (Yamada et al., 2004). Similarly, BrdU incorporates into the DNA of cells undergoing mitosis, and its integration depends on series of factors including the blood brain barrier, extracellular fluid, nucleoside transport mechanism, and pharmacological variables (Cooper-Kuhn and Kuhn, 2002; Liu et al., 2010). However, DCX does not rely on these variables, it detects only newly generated neurons, hence its specificity (Hwang et al., 2008). Therefore, DCX should be regarded as a gold standard marker for detecting neurogenesis.

## DCX mediates cytoskeletal organization by stabilizing the MT

Neuronal migration is a critical step during the development of the nervous system. For a neuron to successfully complete its functions, it must undergo series of cytoskeletal modifications. MTs can modify cell membranes, influence adhesive structures, interact with other cytoskeletal elements, and participate in signaling pathways via different interacting proteins. MTs contribute to various cellular migration processes via its distinct properties coupled with the extension of the MT network throughout the cell cytoplasm. These functions are mediated by MAPs and microtubule-associated motors (Etienne-Manneville, 2013). While MAPs can stabilize MTs against disassembly, they can also influence their dynamics because they are involved in interactions between MTs and other cellular organelles as well as signaling molecules. Typically, DCX is required for the movement of neuroblasts from the neurogenic zones to their point of piquancy. This is achieved by direct interactions with MTs or other neuronal MAPs and by stimulatory signals emanating from several pathways. How does DCX stabilize the MTs and what are the molecular consequences of its interactions with other MAPs?

DCX binds to MTs in migrating cells and as a result promote the movement of these cells (Friocourt et al., 2003; Schaar et al., 2004). It is very important in neurogenesis and is implicated in neuronal migration disorders. Deletion of DCX or its mutation in mice resulted in disorganized (Kappeler et al., 2006; Koizumi et al., 2006a) and retarded movements (Friocourt et al., 2007). Consequently, the leading process of DCX-deficient interneurons branches many times at shorter intervals than normal, but the new branches are very unstable, owing to the fact that cytoskeletal instability in the leading process may prompt neurons to branch more frequently (Kappeler et al., 2006). This suggests that DCX is required to stabilize new leading process branches. Furthermore, the mutations of DCX in human produced a disorganized, unfolded cortex, with band heterotopia, a condition where some neurons remain in cortical white matter making them incapable of reaching the cortex (Kerjan and Gleeson, 2007; Leger et al., 2008; Jaglin and Chelly, 2009), hence the name SBH. However, mutant animals exhibited milder symptoms like seizures, indicating a hippocampal perturbation may be sufficient to generate them, this may be due to the size of the brain of these animals or the redundancy on members of DCX protein family (Nosten-Bertrand et al., 2008; Kerjan et al., 2009). Furthermore, in neurons lacking DCX, dendrites had abnormal appearances and impaired signal transmission across synapses as well as increased pyramidal cell excitability. These factors may underlie the vulnerability of DCX mutant animals to epileptic seizures (Fourniol et al., 2013). At the same time, genetic deletion of DCX produces mice with a heterotopia restricted to the CA3 region of the hippocampus (Kappeler et al., 2006, 2007), which are probably due to structural similarities with other genes in the same family (e.g., DCLK, deputizing in its absence). This disorder becomes more evident when it is combined with mutations in *Lis1* (another gene involved in lissencephaly; Deuel et al., 2006). This evidence point toward catastrophic consequences as a result of DCX mutations, which range from mild to severe neurological disorders, suggesting that DCX play crucial roles in the formation and maintenance of a functional brain.

DCX may directly interact with platelet-activating factor acetylhydrolase IB subunit alpha (PAFAH1B1; also referred to as Lis1), which plays a significant role in regulating the motor protein dynein (Caspi et al., 2000). This interaction was reported to contribute to MT stabilization and enhanced nuclear translocation in neuroblasts and favors the proper development of the cortex. DCX could also serve as a link between MTs and Lis1 to stabilize cellular migration via the phospho-FIGQY motif of neurofascin. Neurofascin is known to play roles in neuronal migration, synaptic plasticity, axonal guidance, as well as neurite outgrowth and fasciculation (Kizhatil et al., 2002; Dijkmans et al., 2010). Co-localization of DCX and neurofascin occurs in migrating neurons and tracts of developing axons. This interaction relies on the phosphorylation of FIGQ-motif of neurofascin and it could be important for MT directional migration of neurons, serving as a regulator of DCX (Table 1). This suggests a role for DCX in cell adhesion (Kizhatil et al., 2002). Although, little is known about the interactions between DCX and neurofascin, existing facts prove that there is more to the functionality of DCX than our current understanding.

**Table 1:** DCX-associated proteins and the function of their interactions.

**Protein/molecule interaction**	**Importance of interactions**	**Molecular site of action**	**Discovery**
PTEN	A major inhibitor of the PI3K/AKT pathway	???	Li et al., 1997, 2003; Endersby and Baker, 2008
PAFAHIB1	Regulates dynein by establishing nucleus-centrosome coupling and increasing MT stabilization as well as nucleation to favor tubulin polymerization.	Direct interaction	Caspi et al., 2000
Tubulin	Enhanced MT polymerization.	First DC domain of DCX	Sapir et al., 2000
Neurofascin	Neuronal migration, neurite outgrowth and fasciculation, as well as synaptic plasticity and axonal guidance	FIGQ-motif of neurofascin	Kizhatil et al., 2002
NeurabinII/spinophilin	Influences the binding of DCX to f-actin and increases affinity for the actin filaments	NuerabinII enhances DCX dephosphorylation at JNK2 phosphorylation sites. Residue Ser 297.	Tsukada et al., 2003, 2005; Coquelle et al., 2006
MARK1 and PRKA	DCX is correctly localized at the leading processes thus MT-binding activity reduces	Ser 447	Schaar et al., 2004
CDK5	Phosphorylates DCX, reduces the amount of DCX that co-localize with MTs, and also reduces the polymerizing effects of DCX	Ser 297	Tanaka et al., 2004
MAPK8/JNK1	Neurite outgrowth of migrating neurons and controlled actin dynamics.	Residue Thr321, Thr331, and Ser334	Gdalyahu et al., 2004
Rai (SHC3/SHCC/ N-SHC)	Mediates signaling pathways leading to GBM invasion.	???	Ortensi et al., 2012
c-Jun N-Terminal Kinase	Regulates neurite extension, decreases DCX affinity for MTs, and promotes cell migration.	Phosphorylates DCX on Ser 332	Jin J. et al., 2010
Kinesin	Transport of JNK signaling module to the neurite tip where JNK then phosphorylate DCX.	Connected to DCX via JIP	Gdalyahu et al., 2004

*???–Interactions that are yet to be determined*.

MT plus-end tracking proteins (+TIPs) are groups of evolutionary conserved cellular factors located at the growing MT plus ends. They regulate MT dynamics by coordinating interactions between MTs and cellular structures as well as signaling factors (Akhmanova and Steinmetz, 2008). End-binding proteins (EB) comprise several +TIPs as they recruit them to MT plus ends in favor of migration (De Groot et al., 2010). DCX is not a +TIP, in fact, it is excluded from the EB domain. It, however, binds to the same site as EBs (Fourniol et al., 2010) and the binding of both molecules to MTs is mutually exclusive but not competitive. Moreover, *in vitro* studies proposed DCX localization to growing MT ends independently of EBs (Bechstedt and Brouhard, 2012; Bechstedt et al., 2014). Recently, Ettinger et al. reported that in the presence of taxanes (spindle poison), DCX preferably binds to depolymerizing MTs, whereas it binds to polymerizing MTs in normal, undisturbed MTs (Ettinger et al., 2016). The interactions between DCX and MTs is so unique that it readily binds to the GTP- or GDP-Pi-bound MT conformation *in vivo*. It is the first anti-+TIP reported to specifically bind to the GDP-MT lattice. Experiments conducted to mimic this type of interactions between DCX and MTs did not result in strong affinity between them (Ettinger et al., 2016). This implies that DCX-MT interaction is required for the stabilization of a growing MT and rescue of depolymerized MTs.

In addition, *Toxoplasma gondii* (*T. gondii*), a member of apicomplexans, possess a cone-shaped feature known as “conoid.” The conoid is made up of 14 helical configurations of fibers. In these organisms, TgDCX, which has a DCX domain has been described as a candidate molecule that dictates the arrangement of conoid fibers. Complete loss of the TgDCX reduced host cell invasion by four-fold compared to wild-type (Nagayasu et al., 2017). Without the DCX domain, the conoid appears irregular, exhibiting stunted growth and its ability to invade host cells consequently diminishes. Hence, the TgDCX has been proposed as an attractive parasite-specific chemotherapeutic target (Nagayasu et al., 2017). Although, it is not regarded as the DCX protein independently, its possesses conserved DCX domain and corresponding functions which are similar to that of the DCX domain found in mammals. This shows that the DCX domains retain its functions across species. These findings further strengthen the idea that DCX, when altered, can be implicated in host cell invasion.

Taken together, these data suggest that DCX stabilizes the MTs and is required for the maintenance of a functional brain. However, a deeper knowledge about the mechanism involved in the process is essential for the rescue of a depolymerizing MT. Meanwhile, it is important to note that these interactions between DCX and MTs are orchestrated by both N-terminus and C-terminus domains. Indeed, no interactions were found in DCX constructs lacking either of the two domains (Kim et al., 2003). This suggested that both domains are equally important in the development of a functional brain.

### MT destabilization may occur by phosphorylation of DCX

**Figure 2:**
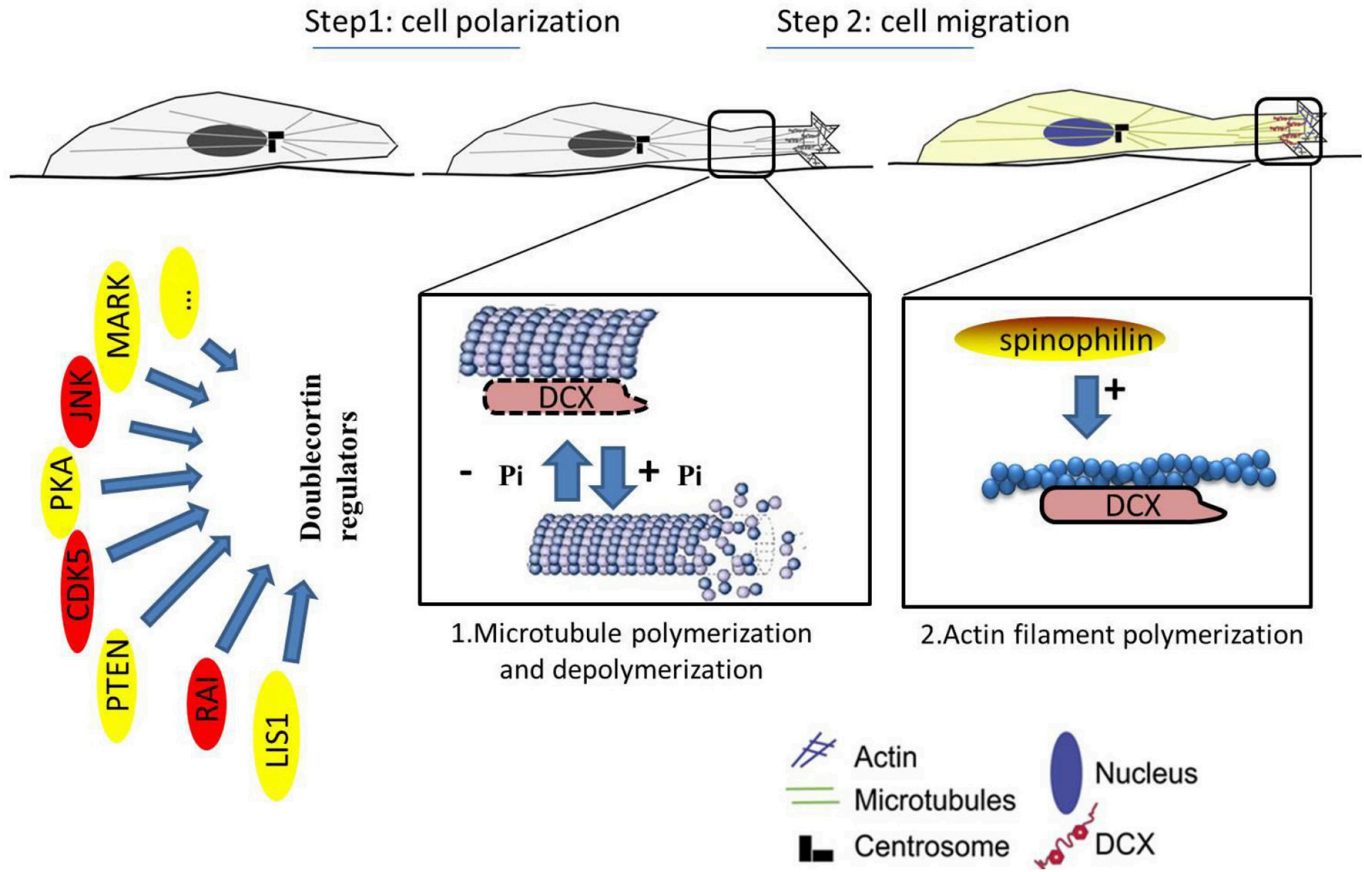
Neuronal migration involves polarization of the cytoskeleton. MTs can induce and/or maintain polarity. Abnormal cell migration likely reflects a faulty mechanism in cell polarization. **1**. Microtubule polymerization and depolymerization (enlarged view of interactions between DCX and MTs). **2**. Actin filament polymerization (enlarged view of the leading process). Reduction in DCX destabilizes MTs *in vitro* leading to polarization impairment. The “doublecortin regulators” act on DCX to aid effective migration. Kinases can phosphorylate DCX on its serine/threonine residues to aid migration of neuroblast, as phosphorylation of DCX is required for localization of the MTs and proper neuronal migration. However, these kinases (in red) are in free-flow in cancer cells and may be carcinogenic. Cancer cells may take advantage of these kinases to destabilize the MTs and promote uncontrolled movement of cells. While DCX is phosphorylated on the MTs, it can also bind to actin filaments at the leading process via spinophilin (gold). This promotes polymerization of actin and improves cellular migration.

MAPs are targets of several protein kinases, and phosphorylation of a MAP determines its activity and localization within the cell. The mechanisms adopted by DCX in stabilizing the MT have been exclusively explained via phosphoregulation as well as its interactions with several MAPs and other cytoskeletal components. It serves as a substrate for some kinases and phosphatases including the protein kinase A (PKA) and microtubule affinity-regulating kinase 1 (MARK1) at residue Ser47 (Schaar et al., 2004), as well as cyclin-dependent kinase 5 (CDK5) mainly at residue Ser297 (Tanaka et al., 2004). MARK1 and PKA- phosphorylation of DCX weakens the DCX-MT interactions as phosphorylation of DCX at Ser47 is required for DCX proteins to be properly localized at the leading processes during neuronal migration. Meanwhile, phosphorylation of CDK5 at Ser297 reduces the amount of DCX that co-assemble with MTs (Marin et al., 2010), a measure of affinity, and decreases the polymerizing effect of DCX, a measure of stabilization. Decreased affinity and stabilizing effects of DCX disrupts the MT, an effect that may lead to a more dynamic MT cytoskeleton (Tanaka et al., 2004; Figure 2). However, further clarification on this event needs to be established to fully understand the functions of DCX.

In addition, DCX can be phosphorylated by mitogen-activated protein kinase 8 (MAPK8/JNK1) at Thr321, Thr331, and Ser334 sites in humans with corresponding sites at Thr326, Thr336, and Ser339 in mouse. Phosphorylation at these sites influences DCX mobilization to the growth cones of the leading processes (Gdalyahu et al., 2004; Jin J. et al., 2010), suggesting that the role of DCX is not restricted to stabilizing the MT but also extended to the actin cytoskeleton. Furthermore, DCX has been reported to interact with actin directly and indirectly through neurabin II (also known as spinophilin; Tsukada et al., 2003).

### DCX interacts with actin filaments

Several reports show that MTs and actin interact with each other to drive cell migration and neuronal growth cone movement (Schaefer et al., 2002). In migrating cells, MTs preferentially bind to and grow along focal adhesion-associated actin bundles (Salmon et al., 2002). Likewise, in neuronal growth cones, filopodial f-actin bundles play a role in MT dynamics as MT subunits polymerize toward the growth cone extension regions of the filopodia (Schaefer et al., 2002). The interactions between MTs and actin may occur either as a regulatory or structural contribution to the mechanics of the cytoskeleton. These interactions are regulated via cytoskeletal-associated proteins which may bind to more than one cytoskeletal component. For instance, MAP2c and MAP1B are known neuron-specific MAPs that interact with the actin cytoskeleton. Interestingly, MAP2c associated with the actin cytoskeleton after phosphorylation and this phosphorylation repressed the binding of MAP2c to the MT (Rodriguez et al., 2003), an interaction similar to the one employed by DCX. The fact that MAP2c dissociates from MTs after its phosphorylation (Ozer and Halpain, 2000) correlates well with reports that the binding of DCX to MTs is negatively regulated by phosphorylation on its serine residues (Schaar et al., 2004; Tanaka et al., 2004). As much as phosphorylation may drive DCX away from the MT cytoskeleton, it may also increase affinity for the actin cytoskeleton. DCX can bind as well as bundle f-actin, this hints at a novel function of DCX.

DCX interacts with actin via spinophilin, an actin-associated protein that acts as a protein phosphatase-1 (PP1)-adaptor. Spinophilin is highly expressed in the dendrites where neurons take up impulses (Allen et al., 1997). When DCX was co-expressed with spinophilin, it interacted with both MTs and f-actin. However, when DCX alone was expressed in cells, it co-localized with MTs but not with f-actin (Tsukada et al., 2003; Yap et al., 2016). Hence, DCX may act as a cross-linking factor to both the actin cytoskeleton and the MT depending on its phosphorylation state with spinophilin. Spinophilin also enhances DCX dephosphorylation at JNK2 phosphorylation sites by upregulating PP1 and this dephosphorylation of DCX regulates its distribution between f-actin and MTs (Tsukada et al., 2005; Bielas et al., 2007). Furthermore, PP1 targets DCX via *PPP1R9B* (the gene encoding neurabin/spinophilin). This leads to dephosphorylation of DCX at Ser297. Dephosphorylation at this site is essential for DCX distribution at the neurite tip during neuronal migration. Moreover, DCX and PPP1R9B co-localize at the axons, where MT and actin interact (Bielas et al., 2007). Studies on the implications of spinophilin mutations may explain the functions of DCX-spinophilin interactions as well as prognosis of some diseases associated with cytoskeletal disorganization.

MAPK8-mediation of DCX is also necessary at the neurite tips during migration (Gdalyahu et al., 2004). When DCX binds to MAPK8-interacting protein 1 (MAPK8IP1/JIP-1), it may indirectly regulate actin dynamics via the RELN pathway. This is possible because JIP-1 directly binds to LRP8 which is an essential component of the pathway (Moon and Wynshaw-Boris, 2013). However, little is known about this regulation. Further to this aspect, more knowledge about these interactions is fundamental to the study of cellular migration.

While neurons are full of MT-stabilizing proteins, DCX has unique properties that cannot be functionally compensated for by other neuronal MAPs (Hoenger and Gross, 2008). Facts presented here show that DCX is not just a MAP but can also function as an actin-associated protein through different molecules to enhance migration of neurons. It is inestimably important in the dynamics and mechanism of the activities of the brain. However, these reports are not enough to fully understand the importance of DCX and cytoskeletal mechanics in the quest to combating abnormalities related to the destabilized cytoskeleton. Therefore, further works on the DCX molecule and its genetic build up should be improved to better address the causes of related diseases.

## Dynamic involvement of DCX in cancers

Studies about cellular dynamics may explain the invasion and metastatic abilities of recurring cancer cells. Several pathways that regulate normal stem cell physiology have been reported to influence tumor cell migration and invasion. Similarly, genes that regulate critical pathways for neural stem cell maintenance, differentiation, and proliferation, are also valuable targets for initiating differentiation as well as invasion of brain tumor stem cells. DCX falls into this category (Das et al., 2008; Ortensi et al., 2013). It is expressed in cancer cells and its contribution to the migration of neuroblasts makes it susceptible to the mechanism involved in the migration and invasion of cancer cells.

DCX is significantly expressed in a variety of cancers even when it is regarded as a neuron-specific MAP. The Human Protein Atlas database reported DCX to be moderately expressed in liver and prostate cancer tissues. 22.2% of prostate cancer tissues reported were positive of DCX as either low or in moderation while 26% of the liver cancer tissues reported were immunopositive of DCX ranging from high to low. In addition, other studies have demonstrated DCX expression in human glioblastomas (GBMs; Daou et al., 2005; Rich et al., 2005; Masui et al., 2008), gangliogliomas (Becker et al., 2002), cortical tubers (Lee et al., 2003), and lissencephaly (Miyata et al., 2004). Interestingly, its expression in brain tumors is concentrated at the invasive front. Consequently, DCX has been used in some studies as a molecular indicator of the infiltrating glioma cells (Bexell et al., 2007; De Rosa et al., 2012). There are existing studies suggesting that the expression of DCX may relatively be considered in cancer prognosis and survival rate. According to Rich et al., osteonectin (SPARC) and semaphorin3B are equally important genes involved in the regulation of cell migration, when combined with DCX, these genes may act as predictive genetic markers for the survival of GBM patients. Simultaneous expression of DCX with SPARC and semaphorin3B indicated a greater risk to patients' survival (Rich et al., 2005), thus proposing a direct link between tumor invasion and patient's survival.

While researches in the past years have mainly been directed toward clarifying how new neurons from the SVZ are generated in response to brain lesions such as cerebral ischemia, epileptic seizures, and mechanical trauma, much less is known about the SVZ's response to the growth of a malignant brain tumor. It may be that cells migrating from the neurogenic zones respond to inflammation generated by cancers cells. Further studies are needed to evaluate this concept with major focus on DCX.

### DCX activities in the normal cells may be implicated in cancer cells

Several kinases and phosphatases involved in the phosphorylation and dephosphorylation of DCX have been implicated in brain tumor proliferation and invasion. Kinases and phosphatases are frequently deregulated in GBMs, therefore suggesting possible mechanisms favoring the invasive abilities of the cancer cells (Ortensi et al., 2013). DCX is phosphorylated by CDK5 on Ser297 residue (Tanaka et al., 2004), likewise on Ser47 by PKA and MARK (Schaar et al., 2004), both eventually led to reduction in DCX's binding affinity for MTs and in turn enhancing migration, a mechanism that can be implicated in the migration and invasion of highly invasive glioma (Figure 2). On the other hand, c-Jun N-terminal kinase (JNK) phosphorylation of DCX at Ser332 decreased the affinity of DCX for tubulin, thus cell migration was enhanced (Jin J. et al., 2010). Perhaps these phosphorylations may be required to initiate and maintain uncontrolled movement of cancer cells due to the destabilization of the MTs, as uncooperative MT stapling and aversion for MT tips may explain DCX involvement in the movement of invasive cancer cells.

Moreover, phosphatase and tensin homolog (PTEN), an antioncogene often expressed in human cancer act on DCX. From the STRING database (functional protein association networks), DCX was found to interact with PTEN directly via AP-1 (Jun), a product of c-Jun. PTEN has also been reported to act as a suppressor of the PI3K/AKT pathway and is often deleted in GBMs (Endersby and Baker, 2008). We suspect existing links between these two and there might be a correlation between PTEN and DCX in GBM invasion. In addition, Rai (SHC3/SHCC/N-SHC) is a neuron-specific member of the family of Shc-like adaptor proteins. It is expressed in neurons, in response to ischemic damage to confer neuroprotective responses via the activation of the PI3K/Akt pathway, leading to cell survival (Ortensi et al., 2012). In an *in vivo* and *ex vivo* experiment, Ortensi et al. demonstrated that silencing Rai in cancer stem/progenitor cells derived from GBM patients reduced cell dynamics and their ability to invade normal cells. Rai also mediates different signaling pathways which eventually leads to metalloproteinase upregulation and GBM invasion. DCX is a target of this adaptor protein in NSCs and in CSCs derived from GBM patients. Moreover, some cells that express Rai reside predominantly in the tumor invasion front. These cells also express DCX and OLIG2 which are indicators of adult neurogenesis (Ortensi et al., 2012).

Meanwhile, despite reports from several types of research confirming DCX expression in cancer cells (Bexell et al., 2007; De Rosa et al., 2012), Santra et al. frequently reported that DCX is absent in glioma cell lines (Santra et al., 2011). However, this conflicting observation may be due to the type of cell line used (U87 cell-line), since the experiment was only carried out using a single cell line. On the other hand, experiments from De Rosa et al. confirmed the expression of DCX in both primary and recurrent GBM tumors derived from patients. Therefore, it would be peremptory to conclude that DCX is not expressed in brain tumors based on reports from Santra et al. Interestingly, they also reported that DCX serves as a tumor suppressor gene. When exogenous DCX was added to cells, reduced proliferation and diminished stem cell renewal were observed (Santra et al., 2010), this is also an interesting development that needs to be validated. Experiments designed to alter the gene expression with different technologies is needed to support this observation in different cell lines and animal models. However, there must be caution on how the genetic expression resulting from gene alteration is translated for accurate applications.

Even though there are several reports about the regulation of DCX during neurogenesis, there is more to be explored regarding the initiation and regulation of brain cancers. This may lead to the discovery of new pharmaceutical targets and hence aid effective treatment and eradication of malignant tumor cells.

### DCX may be an independent biomarker for NB

Neuroblastoma (NB) is the most common cancer occurring from early childhood. It is responsible for a significant number of cancer deaths in children. It is characterized by poor prognosis in infants diagnosed at an early stage of life and metastasize to various organs of the body (Esposito et al., 2017). In metastatic NB patients, accurate risk stratification and disease monitoring would reduce relapse probabilities. Reverse transcriptase quantitative polymerase chain reaction (RT-qPCR) for NB mRNAs in bone marrow (BM) or peripheral blood (PB) can be predictive of outcome (Viprey et al., 2014). DCX has been identified as a sensitive and specific minimal residual disease biomarker (Viprey et al., 2014). Yanez et al. reported high levels of DCX mRNA detection by RT-qPCR in the PB and BM at diagnosis. According to them, high levels of DCX in BM independently predicted poor event-free survival and overall survival in NB patients (Yanez et al., 2016). They consequently suggested that DCX mRNA levels in PB and BM assessed by RT-qPCR may be considered in new pretreatment risk stratification strategies to reliably estimate outcome differences in metastatic NB patients. This may further enhance the mode of stratifying NB patients into risk groups for effective and proper treatment. However, this report alone is insufficient to support these claims since Yanez et al. did not include low and intermediate NB patients during diagnosis. Therefore, further research involving these sets of patients is required to validate the accuracy of this technique.

## The DCX superfamily; sharing attributes from appearance to behaviors

DCX superfamily members may exhibit partial functional co-operation during adult neurogenesis. Genetic studies and detailed sequence analysis of mutants of the DCX family members showed that genes that encode this family member may co-operate with each other in an organized manner during neurodevelopment (Deuel et al., 2006). This offers a platform for the mapping of tandem repeats located in conserved DCX domains (Sapir et al., 2000). The sequence analysis revealed that DCX is built from an N-terminal tandem of homologous 90-amino-acid (11 kDa) domains which were consequently named DC domains. These domains are separated by a well-conserved but presumed unstructured linker and are followed by a C-terminal serine/proline-rich domain. Studies further reported that the first DCX domain binds tubulin and promotes polymerization (Fourniol et al., 2013).

DCX superfamily members are MAPs that participate in cytoskeletal stabilization, consequently promoting cellular dynamics in immature neurons (Koizumi et al., 2006b). Analysis of loss-of-function mutations in DCX family members (DCX, DCLK, or DCLK2) showed that mutations in a single member of this family resulted in less severe developmental defects compared to multiple mutations across the family (Deuel et al., 2006; Koizumi et al., 2006b; Kerjan et al., 2009). Defects in combined mutations of the DCX superfamily members range from abnormal hippocampus to perinatal lethality (Deuel et al., 2006; Koizumi et al., 2006b).

### Loss of DCX can be compensated for to some extent by DCLK

**Figure 3:**
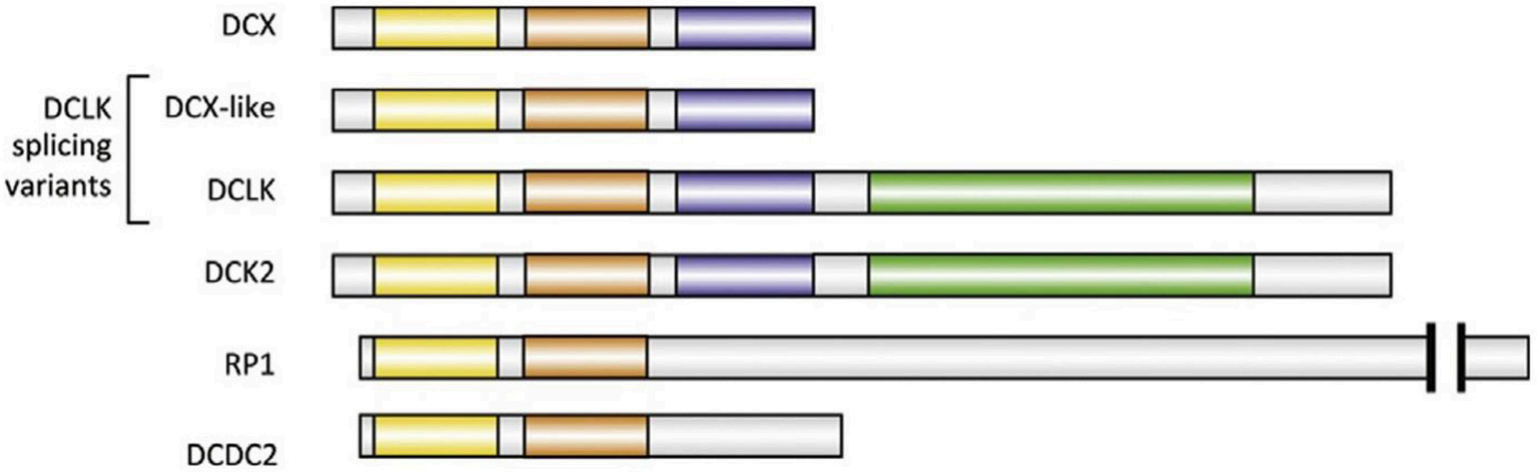
Major human paralogs of DCX. All paralogs illustrated here have two DC domains (N-DC depicted in yellow, C-DC in orange). Two of the four isoforms of DCLK are represented: DCX-like and full-length DCLK, which has a kinase domain (green). DCLK full-length has a very close homolog called DCK2. The serine/proline-rich domain is colored blue (Fourniol et al., 2013).

Doublecortin-like kinase (DCLK), initially known as doublecortin and CaM kinase-like 1 is the closest DCX paralog (Burgess and Reiner, 2000). It has several splicing isoforms, with a DCX-like isoform that is 72% identical to DCX. DCLK is a 729-amino-acid protein, with a C-terminal serine/threonine–protein kinase domain, similar to CaM kinase II (Koizumi et al., 2006b; Figure 3). Indications about the potential functions of DCX and DCLK are hinted by their distribution in the growing axons. DCX is enriched especially in the growth cones of elongating axons (Friocourt et al., 2003; Schaar et al., 2004). It associates in a gradient along MTs that increase precipitously as they extend from the base of the growth cone to its periphery (Tint et al., 2009; Jean et al., 2012). DCLK display similar distribution, suggesting that both proteins have specialized roles on MTs for the radically unique environment of the growth cone compared with the axonal shaft (Burgess and Reiner, 2000; Jean et al., 2012). Clues about the potential functions of DCX and DCLK are suggested by their distribution in growing axons. Targeted deletion of DCX in mice produces mild phenotypes at least to an extent (Deuel et al., 2006). DCX binds between the pfs of MTs which may be conducive to enable the MTs maintain a relatively straight conformation (Moores et al., 2004). There are existing reports that no distinct phenotype was observed in neurons when DCX alone was downregulated (Tint et al., 2009). Neither the growth cone morphology nor the MT distribution appeared disrupted (Tint et al., 2009; Jean et al., 2012). Furthermore, similar observations were made in neurons lacking DCLK (Deuel et al., 2006).

Interestingly, knocking out both DCX and DCLK simultaneously, presented severe symptoms with frequent seizures and irregular lamination of the hippocampus (Deuel et al., 2006; Koizumi et al., 2006b). The severity of disorders related to DCX mutations was reportedly minimal due to genetically redundant pathways involving DCLK. Either molecule can compensate the absence of the other to a large extent in single knockout out experiment (Jean et al., 2012). When DCX and DCLK are rightly bound to MTs, they provide support and strengthen the arrangement of tubulin. This is possible because both proteins bind to the MTs at the joint between two pfs (the weakest point on the MT). Moreover, the binding prevents MTs from folding, thus inhibiting MT depolymerization (Moores et al., 2004). Like the DCX, DCLK should also be given a close attention as it is the closest member of the DCX family whose functions have been reported to be similar to that of DCX, more than any other neuronal MAP. Also, more studies on DCX in DCLK knockout animal models will give a clearer insight into the remaining features of DCX that are yet to be discovered.

### DCDC2

Another DCX superfamily member that possess similar conformation and display partial redundancy to DCX is the DCX domain-containing protein 2 (DCDC2), encoded by the *DCDC2* gene. It is implicated in developmental dyslexia also known as the reading disorder (Lind et al., 2010). DCDC2 is involved in speech processing in the human brain while writing (Marino et al., 2012). Like its family members, DCDC2 has two DCX domains (Figure 3), which have been reported to bind to MTs and promote tubulin polymerization (Grammatikopoulos et al., 2016). Furthermore, DCDC2 is reportedly found in the hippocampal neurons, at the neuronal cilia where it modifies cilia signaling. Indeed, its protein product is involved in the control of activity and size of primary cilia in neurons (Massinen et al., 2011). The *DCDC2* gene expression is frequently found in the neocortex of immature rodents as well as in human neocortex (Burbridge et al., 2008). Altering DCDC2 expression in neurons of the neocortex of immature rats resulted in the irregular migration of the neurons. This indicated that DCDC2 facilitates neuronal development, further clarifying the proximity at which the DCX superfamily members function (Burbridge et al., 2008). Moreover, DCDC2 may be involved in cellular migration due to its interaction with several cytoskeletal-related proteins like its family members (Reiner et al., 2006). Additionally, mutant mice with DCX RNAi combined with DCDC2 knockout exhibited more severe abnormality compared with just DCX knockout mice, in this case, the dendrites appeared irregular (Wang Y. et al., 2011). Hence, DCDC2 is required during neurogenesis and may be involved in cellular mobility.

### RP1

DCX paralogs also include oxygen-regulated protein 1 (RP1), a protein that is mutated in retinitis pigmentosa (a common form of inherited blindness) and whose MT-stabilizing activity is essential for photoreceptor cell development (Liu et al., 2004). Interestingly, erroneous eye receptor development as a result of RP1 mutation is associated with reduced JNK signaling (Liu et al., 2005), suggesting an interaction between RP1 and the JNK pathway (Dijkmans et al., 2010). This protein is specifically expressed in photoreceptors because of its DCX-domain (Liu et al., 2002).

## Conclusion

In this review, we have assembled the features of DCX and highlighted its importance in the CNS as well as cancers to identify research gaps and project new research areas for the benefit of cellular dynamics and neurogenesis. DCX is a decisive factor in the development of neuroblasts. It orchestrates cellular dynamics during movements from neurogenic zones to the region of lesions. While it is generally known to be involved in lissencephaly and SBH, it is also very important in the development of cancer cells. Newly generated cells migrating from the neurogenic zones are DCX positive, and mutations in the DCX protein would lead to migration disorders causing lissencephaly and SBH. Hence, DCX has been adopted as a marker of neurogenesis in the SVZ and SGZ.

DCX is a neuronal MAP that is crucial to the migration of neuroblasts, and this makes it a vulnerable target for cancer cells. In other words, its original function may be overridden by cancer cells, functioning in a two-way system in favor of cancer cells to invade normal cells. The data reviewed above suggest that DCX regulates cancer cells mobility and invasion of adjacent tissues by remodeling the cytoskeletal network in response to signals influencing the survival of cancer cells. When DCX is phosphorylated, MTs unbundles and depolymerization sets in, a process that may be adopted by migrating cancer cells, taking advantage of the destabilized MT network to invade its surrounding cells. It also associates with actin filaments via an actin-associated protein, spinophilin. When DCX is phosphorylated on its serine residues, it tends to leave the MTs and shifts toward f-actin to aid the polymerization of the actin filaments and improve migration, another process that may be implicated in the migration of cancer cells. DCX can also serve as a marker for cancer prognosis. When combined with other genes can be used as genetic markers of GBM survival; the higher the expression, the lower the chances of survival. Furthermore, the levels of DCX mRNA in PB and BM can also be considered as a novel marker for the stratification of NB patients into risk groups to improve accuracy in treatment.

Despite efforts made to unravel the mechanisms that underlie cell migration, a lot more remain unknown. Thus, finding different pathways through which DCX conjugates with other proteins to stabilize the MT and its activities in the actin cytoskeleton should be prioritized as these pathways may provide insights for inhibiting tumor progression. What effect does DCX have on the proliferation capacity of brain tumors? So far, only a few reports can be found about the protective or destructive effects of DCX in cancer cells. These knowledge gaps suggest a possible path to future research in the quest to combating cancer recurrence and invasion. During the past few years, we noticed a decrease in research about DCX, this is a major concern as we believe there is more to be explored in this field, most especially its roles in cancer cell initiation. Besides its expression in the CNS, a recent study has confirmed the expression of DCX in the cytoplasm of beta cells and has been postulated as a possible biomarker for beta-cell injury. This expands the functional scope of DCX and calls for further research into the relationship between neurogenesis and the production of new beta cells in response to diabetes. It is predictable that the importance of DCX in cancer cells will be further appreciated with a wide interest in its research by scientists from different fields and the development of new therapeutic agents directed against the cytoskeleton to curb the development of cancer cells.

## Author contributions

AA is the primary first author and YX is the co-first author of this review article, while DG is the corresponding author. YG, GJ, CT, and ZA also contributed to the write up of the review in the above order in a significant way. All the authors are from Xuzhou Medical College recently renamed to Xuzhou Medical University.

### Conflict of interest statement

The authors declare that the research was conducted in the absence of any commercial or financial relationships that could be construed as a potential conflict of interest.

## References

[B1] Akhmanova A. Steinmetz M. O. (2008). Tracking the ends, a dynamic protein network controls the fate of microtubule tips. Nat. Rev. Mol. Cell Biol. 9, 309–322. 10.1038/nrm2369 18322465

[B2] Allen P. B. Ouimet C. C. Greengard P. (1997). Spinophilin, a novel protein phosphatase 1 binding protein localized to dendritic spines. Proc. Natl. Acad. Sci. U.S.A. 94, 9956–9961. 10.1073/pnas.94.18.9956 9275233 PMC23308

[B3] Amrein I. Dechmann D. K. Winter Y. Lipp H. P. (2007). Absent or low rate of adult neurogenesis in the hippocampus of bats (Chiroptera). PLoS ONE 2:e455. 10.1371/journal.pone.0000455 17520014 PMC1866182

[B4] Balthazart J. Boseret G. Konkle A. T. Hurley L. L. Ball G. F. (2008). Doublecortin as a marker of adult neuroplasticity in the canary song control nucleus HVC. Eur. J. Neurosci. 27, 801–817. 10.1111/j.1460-9568.2008.06059.x 18333960

[B5] Batailler M. Droguerre M. Baroncini M. Fontaine C. Prevot V. Migaud M. (2014). DCX-expressing cells in the vicinity of the hypothalamic neurogenic niche, a comparative study between mouse, sheep, and human tissues. J. Comp. Neurol. 522, 1966–1985. 10.1002/cne.23514 24288185

[B6] Bechstedt S. Brouhard G. J. (2012). Doublecortin recognizes the 13-protofilament microtubule cooperatively and tracks microtubule ends. Dev. Cell 23, 181–192. 10.1016/j.devcel.2012.05.006 22727374 PMC3951992

[B7] Bechstedt S. Lu K. Brouhard G. J. (2014). Doublecortin recognizes the longitudinal curvature of the microtubule end and lattice. Curr. Biol. 24, 2366–2375. 10.1016/j.cub.2014.08.039 25283777

[B8] Becker A. J. Klein H. Baden T. Aigner L. Normann S. Elger C. E.. (2002). Mutational and expression analysis of the reelin pathway components CDK5 and doublecortin in gangliogliomas. Acta Neuropathol. 104, 403–408. 10.1007/s00401-002-0570-4 12200628

[B9] Begg D. P. Woods S. C. (2013). Interactions between the central nervous system and pancreatic islet secretions, a historical perspective. Adv. Physiol. Educ. 37, 53–60. 10.1152/advan.00167.2012 23471249 PMC3776474

[B10] Bexell D. Gunnarsson S. Nordquist J. Bengzon J. (2007). Characterization of the subventricular zone neurogenic response to rat malignant brain tumors. Neuroscience 147, 824–832. 10.1016/j.neuroscience.2007.04.058 17583435

[B11] Bielas S. L. Serneo F. F. Chechlacz M. Deerinck T. J. Perkins G. A. Allen P. B.. (2007). Spinophilin facilitates dephosphorylation of doublecortin by PP1 to mediate microtubule bundling at the axonal wrist. Cell 129, 579–591. 10.1016/j.cell.2007.03.023 17482550 PMC1920181

[B12] Brown J. P. Couillard-Despres S. Cooper-Kuhn C. M. Winkler J. Aigner L. Kuhn H. G. (2003). Transient expression of doublecortin during adult neurogenesis. J. Comp. Neurol. 467, 1–10. 10.1002/cne.10874 14574675

[B13] Burbridge T. J. Wang Y. Volz A. J. Peschansky V. J. Lisann L. Galaburda A. M.. (2008). Postnatal analysis of the effect of embryonic knockdown and overexpression of candidate dyslexia susceptibility gene homolog Dcdc2 in the rat. Neuroscience 152, 723–733. 10.1016/j.neuroscience.2008.01.020 18313856 PMC2424111

[B14] Burgess H. A. Reiner O. (2000). Doublecortin-like kinase is associated with microtubules in neuronal growth cones. Mol. Cell. Neurosci. 16, 529–541. 10.1006/mcne.2000.0891 11083916

[B15] Caspi M. Atlas R. Kantor A. Sapir T. Reiner O. (2000). Interaction between LIS1 and doublecortin, two lissencephaly gene products. Hum. Mol. Genet. 9, 2205–2213. 10.1093/oxfordjournals.hmg.a018911 11001923

[B16] Chawana R. Alagaili A. Patzke N. Spocter M. A. Mohammed O. B. Kaswera C.. (2014). Microbats appear to have adult hippocampal neurogenesis, but post-capture stress causes a rapid decline in the number of neurons expressing doublecortin. Neuroscience 277, 724–733. 10.1016/j.neuroscience.2014.07.063 25106130

[B17] Cooper-Kuhn C. M. Kuhn H. G. (2002). Is it all DNA repair? Methodological considerations for detecting neurogenesis in the adult brain. Brain Res. Dev. Brain Res. 134, 13–21. 10.1016/S0165-3806(01)00243-7 11947933

[B18] Coquelle F. M. Levy T. Bergmann S. Wolf S. G. Bar-El D. Sapir T.. (2006). Common and divergent roles for members of the mouse DCX superfamily. Cell Cycle 5, 976–983. 10.4161/cc.5.9.2715 16628014

[B19] Couillard-Despres S. Winner B. Karl C. Lindemann G. Schmid P. Aigner R.. (2006). Targeted transgene expression in neuronal precursors, watching young neurons in the old brain. Eur. J. Neurosci. 24, 1535–1545. 10.1111/j.1460-9568.2006.05039.x 17004917

[B20] Couillard-Despres S. Winner B. Schaubeck S. Aigner R. Vroemen M. Weidner N.. (2005). Doublecortin expression levels in adult brain reflect neurogenesis. Eur. J. Neurosci. 21, 1–14. 10.1111/j.1460-9568.2004.03813.x 15654838

[B21] Daou M. C. Smith T. W. Litofsky N. S. Hsieh C. C. Ross A. H. (2005). Doublecortin is preferentially expressed in invasive human brain tumors. Acta Neuropathol. 110, 472–480. 10.1007/s00401-005-1070-0 16195916

[B22] Das S. Srikanth M. Kessler J. A. (2008). Cancer stem cells and glioma. Nat. Clin. Pract. Neurol. 4, 427–435. 10.1038/ncpneuro0862 18628751

[B23] De Groot C. O. Jelesarov I. Damberger F. F. Bjelic S. Scharer M. A. Bhavesh N. S.. (2010). Molecular insights into mammalian end-binding protein heterodimerization. J. Biol. Chem. 285, 5802–5814. 10.1074/jbc.M109.068130 20008324 PMC2820806

[B24] De Nevi E. Marco-Salazar P. Fondevila D. Blasco E. Perez L. Pumarola M. (2013). Immunohistochemical study of doublecortin and nucleostemin in canine brain. Eur. J. Histochem. 57:e9. 10.4081/ejh.2013.e9 23549468 PMC3683616

[B25] De Rosa A. Pellegatta S. Rossi M. Tunici P. Magnoni L. Speranza M. C.. (2012). A radial glia gene marker, fatty acid binding protein 7 (FABP7), is involved in proliferation and invasion of glioblastoma cells. PLoS ONE 7:e52113. 10.1371/journal.pone.0052113 23284888 PMC3528762

[B26] Deuel T. A. Liu J. S. Corbo J. C. Yoo S. Y. Rorke-Adams L. B. Walsh C. A. (2006). Genetic interactions between doublecortin and doublecortin-like kinase in neuronal migration and axon outgrowth. Neuron 49, 41–53. 10.1016/j.neuron.2005.10.038 16387638

[B27] Dijkmans T. F. van Hooijdonk L. W. Fitzsimons C. P. Vreugdenhil E. (2010). The doublecortin gene family and disorders of neuronal structure. Cent. Nerv. Syst. Agents Med. Chem. 10, 32–46. 10.2174/187152410790780118 20236041

[B28] Endersby R. Baker S. J. (2008). PTEN signaling in brain, neuropathology and tumorigenesis. Oncogene 27, 5416–5430. 10.1038/onc.2008.239 18794877

[B29] Engelhardt M. Bogdahn U. Aigner L. (2005). Adult retinal pigment epithelium cells express neural progenitor properties and the neuronal precursor protein doublecortin. Brain Res. 1040, 98–111. 10.1016/j.brainres.2005.01.075 15804431

[B30] Esposito M. R. Aveic S. Seydel A. Tonini G. P. (2017). Neuroblastoma treatment in the post-genomic era. J. Biomed. Sci. 24, 14. 10.1186/s12929-017-0319-y 28178969 PMC5299732

[B31] Etienne-Manneville S. (2013). Microtubules in cell migration. Annu. Rev. Cell Dev. Biol. 29, 471–499. 10.1146/annurev-cellbio-101011-155711 23875648

[B32] Ettinger A. van Haren J. Ribeiro S. A. Wittmann T. (2016). Doublecortin is excluded from growing microtubule ends and recognizes the GDP-Microtubule Lattice. Curr. Biol. 26, 1549–1555. 10.1016/j.cub.2016.04.020 27238282 PMC5023073

[B33] Feng Y. Walsh C. A. (2001). Protein-protein interactions, cytoskeletal regulation and neuronal migration. Nat. Rev. Neurosci. 2, 408–416. 10.1038/35077559 11389474

[B34] Fernandez-Lopez B. Romaus-Sanjurjo D. Senra-Martinez P. Anadon R. Barreiro-Iglesias A. Rodicio M. C. (2016). Spatiotemporal Pattern of Doublecortin Expression in the Retina of the Sea Lamprey. Front. Neuroanat. 10:5. 10.3389/fnana.2016.00005 26858609 PMC4731500

[B35] Fourniol F. J. Sindelar C. V. Amigues B. Clare D. K. Thomas G. Perderiset M.. (2010). Template-free 13-protofilament microtubule-MAP assembly visualized at 8 A resolution. J. Cell Biol. 191, 463–470. 10.1083/jcb.201007081 20974813 PMC3003314

[B36] Fourniol F. Perderiset M. Houdusse A. Moores C. (2013). Structural studies of the doublecortin family of MAPs. Methods Cell Biol. 115, 27–48. 10.1016/B978-0-12-407757-7.00003-7 23973064

[B37] Fowler C. D. Johnson F. Wang Z. (2005). Estrogen regulation of cell proliferation and distribution of estrogen receptor-alpha in the brains of adult female prairie and meadow voles. J. Comp. Neurol. 489, 166–179. 10.1002/cne.20638 15984004 PMC3962047

[B38] Fox R. A. Roth T. C. II. LaDage L. D. Pravosudov V. V. (2010). No effect of social group composition or size on hippocampal formation morphology and neurogenesis in mountain chickadees (*Poecile gambeli*). Dev. Neurobiol. 70, 538–547. 10.1002/dneu.20795 20336697 PMC2913135

[B39] Friocourt G. Koulakoff A. Chafey P. Boucher D. Fauchereau F. Chelly J.. (2003). Doublecortin functions at the extremities of growing neuronal processes. Cereb. Cortex 13, 620–626. 10.1093/cercor/13.6.620 12764037

[B40] Friocourt G. Liu J. S. Antypa M. Rakic S. Walsh C. A. Parnavelas J. G. (2007). Both doublecortin and doublecortin-like kinase play a role in cortical interneuron migration. J. Neurosci. 27, 3875–3883. 10.1523/JNEUROSCI.4530-06.2007 17409252 PMC6672408

[B41] Gdalyahu A. Ghosh I. Levy T. Sapir T. Sapoznik S. Fishler Y.. (2004). DCX, a new mediator of the JNK pathway. EMBO J. 23, 823–832. 10.1038/sj.emboj.7600079 14765123 PMC380994

[B42] Gleeson J. G. (2000). Classical lissencephaly and double cortex (subcortical band heterotopia), LIS1 and doublecortin. Curr. Opin. Neurol. 13, 121–125. 10.1097/00019052-200004000-00002 10987567

[B43] Gleeson J. G. Allen K. M. Fox J. W. Lamperti E. D. Berkovic S. Scheffer I.. (1998). Doublecortin, a brain-specific gene mutated in human X-linked lissencephaly and double cortex syndrome, encodes a putative signaling protein. Cell 92, 63–72. 10.1016/S0092-8674(00)80899-5 9489700

[B44] Gleeson J. G. Lin P. T. Flanagan L. A. Walsh C. A. (1999). Doublecortin is a microtubule-associated protein and is expressed widely by migrating neurons. Neuron 23, 257–271. 10.1016/S0896-6273(00)80778-3 10399933

[B45] Grammatikopoulos T. Sambrotta M. Strautnieks S. Foskett P. Knisely A. S. Wagner B.. (2016). Mutations in DCDC2 (doublecortin domain containing protein 2) in neonatal sclerosing cholangitis. J. Hepatol. 65, 1179–1187. 10.1016/j.jhep.2016.07.017 27469900 PMC5116266

[B46] Hall Z. J. Macdougall-Shackleton S. A. (2012). Influence of testosterone metabolites on song-control system neuroplasticity during photostimulation in adult European starlings (*Sturnus vulgaris*). PLoS ONE 7:e40060. 10.1371/journal.pone.0040060 22792214 PMC3391231

[B47] Hoenger A. Gross H. (2008). Structural investigations into microtubule-MAP complexes. Methods Cell Biol. 84, 425–444. 10.1016/S0091-679X(07)84014-3 17964939

[B48] Hwang I. K. Yoo K. Y. Yi S. S. Kwon Y. G. Ahn Y. K. Seong J. K.. (2008). Age-related differentiation in newly generated DCX immunoreactive neurons in the subgranular zone of the gerbil dentate gyrus. Neurochem. Res. 33, 867–872. 10.1007/s11064-007-9528-1 17987384

[B49] Itoh Y. Kampf K. Arnold A. P. (2006). Assignment of human X-linked genes to a zebra finch microchromosome by *in situ* hybridization of BAC clones. Cytogenet. Genome Res. 112, 342M. 10.1159/000089903 16484804

[B50] Jaglin X. H. Chelly J. (2009). Tubulin-related cortical dysgeneses, microtubule dysfunction underlying neuronal migration defects. Trends Genet. 25, 555–566. 10.1016/j.tig.2009.10.003 19864038

[B51] Jean D. C. Baas P. W. Black M. M. (2012). A novel role for doublecortin and doublecortin-like kinase in regulating growth cone microtubules. Hum. Mol. Genet. 21, 5511–5527. 10.1093/hmg/dds395 23001563 PMC3516135

[B52] Jiang L. Brackeva B. Stange G. Verhaeghen K. Costa O. Couillard-Despres S.. (2013). LC-MS/MS identification of doublecortin as abundant beta cell-selective protein discharged by damaged beta cells *in vitro*. J. Proteomics 80, 268–280. 10.1016/j.jprot.2012.12.031 23337804

[B53] Jin K. Wang X. Xie L. Mao X. O. Greenberg D. A. (2010). Transgenic ablation of doublecortin-expressing cells suppresses adult neurogenesis and worsens stroke outcome in mice. Proc. Natl. Acad. Sci. U.S.A. 107, 7993–7998. 10.1073/pnas.1000154107 20385829 PMC2867852

[B54] Jin J. Suzuki H. Hirai S. Mikoshiba K. Ohshima T. (2010). JNK phosphorylates Ser332 of doublecortin and regulates its function in neurite extension and neuronal migration. Dev. Neurobiol. 70, 929–942. 10.1002/dneu.20833 20715151

[B55] Kappeler C. Dhenain M. Phan Dinh Tuy F. Saillour Y. Marty S. Fallet-Bianco C.. (2007). Magnetic resonance imaging and histological studies of corpus callosal and hippocampal abnormalities linked to doublecortin deficiency. J. Comp. Neurol. 500, 239–254. 10.1002/cne.21170 17111359

[B56] Kappeler C. Saillour Y. Baudoin J. P. Tuy F. P. Alvarez C. Houbron C.. (2006). Branching and nucleokinesis defects in migrating interneurons derived from doublecortin knockout mice. Hum. Mol. Genet. 15, 1387–1400. 10.1093/hmg/ddl062 16571605

[B57] Kerjan G. Gleeson J. G. (2007). Genetic mechanisms underlying abnormal neuronal migration in classical lissencephaly. Trends Genet. 23, 623–630. 10.1016/j.tig.2007.09.003 17997185

[B58] Kerjan G. Koizumi H. Han E. B. Dube C. M. Djakovic S. N. Patrick G. N.. (2009). Mice lacking doublecortin and doublecortin-like kinase 2 display altered hippocampal neuronal maturation and spontaneous seizures. Proc. Natl. Acad. Sci. U.S.A. 106, 6766–6771. 10.1073/pnas.0812687106 19342486 PMC2672532

[B59] Kim M. H. Cierpicki T. Derewenda U. Krowarsch D. Feng Y. Devedjiev Y.. (2003). The DCX-domain tandems of doublecortin and doublecortin-like kinase. Nat. Struct. Biol. 10, 324–333. 10.1038/nsb918 12692530

[B60] Kim Y. O. Nam T. S. Park C. Kim S. K. Yoon W. Choi S. Y.. (2016). Familial pachygyria in both genders related to a DCX mutation. Brain Dev. 38, 585–589. 10.1016/j.braindev.2015.12.005 26743950

[B61] Kim Y.-H. Sun W. (2012). Distribution of doublecortin immunoreactivities in developing chick retina. Appl. Microscopy 42, 142–146. 10.9729/AM.2012.42.3.142

[B62] Kizhatil K. Wu Y. X. Sen A. Bennett V. (2002). A new activity of doublecortin in recognition of the phospho-FIGQY tyrosine in the cytoplasmic domain of neurofascin. J. Neurosci. 22, 7948–7958. 12223548 10.1523/JNEUROSCI.22-18-07948.2002 PMC6758080

[B63] Koizumi H. Higginbotham H. Poon T. Tanaka T. Brinkman B. C. Gleeson J. G. (2006a). Doublecortin maintains bipolar shape and nuclear translocation during migration in the adult forebrain. Nat. Neurosci. 9, 779–786. 10.1038/nn1704 16699506

[B64] Koizumi H. Tanaka T. Gleeson J. G. (2006b). Doublecortin-like kinase functions with doublecortin to mediate fiber tract decussation and neuronal migration. Neuron 49, 55–66. 10.1016/j.neuron.2005.10.040 16387639

[B65] LaDage L. D. Roth T. C. II. Fox R. A. Pravosudov V. V. (2010). Ecologically relevant spatial memory use modulates hippocampal neurogenesis. Proc. Biol. Sci. 277, 1071–1079. 10.1098/rspb.2009.1769 19939840 PMC2842758

[B66] Lee A. Maldonado M. Baybis M. Walsh C. A. Scheithauer B. Yeung R.. (2003). Markers of cellular proliferation are expressed in cortical tubers. Ann. Neurol. 53, 668–673. 10.1002/ana.10579 12731003

[B67] Leger P. L. Souville I. Boddaert N. Elie C. Pinard J. M. Plouin P.. (2008). The location of DCX mutations predicts malformation severity in X-linked lissencephaly. Neurogenetics 9, 277–285. 10.1007/s10048-008-0141-5 18685874

[B68] Li J. Yen C. Liaw D. Podsypanina K. Bose S. Wang S. I.. (1997). PTEN, a putative protein tyrosine phosphatase gene mutated in human brain, breast, and prostate cancer. Science 275, 1943–1947. 10.1126/science.275.5308.1943 9072974

[B69] Li L. He F. Litofsky N. S. Recht L. D. Ross A. H. (2003). Profiling of genes expressed by PTEN haploinsufficient neural precursor cells. Mol. Cell. Neurosci. 24, 1051–1061. 10.1016/j.mcn.2003.08.010 14697668

[B70] Lind P. A. Luciano M. Wright M. J. Montgomery G. W. Martin N. G. Bates T. C. (2010). Dyslexia and DCDC2, normal variation in reading and spelling is associated with DCDC2 polymorphisms in an Australian population sample. Eur. J. Hum. Genet. 18, 668–673. 10.1038/ejhg.2009.237 20068590 PMC2987340

[B71] Liu D. Z. Ander B. P. Xu H. Shen Y. Kaur P. Deng W.. (2010). Blood-brain barrier breakdown and repair by Src after thrombin-induced injury. Ann. Neurol. 67, 526–533. 10.1002/ana.21924 20437588 PMC2919346

[B72] Liu J. Huang Q. Higdon J. Liu W. Xie T. Yamashita T.. (2005). Distinct gene expression profiles and reduced JNK signaling in retinitis pigmentosa caused by RP1 mutations. Hum. Mol. Genet. 14, 2945–2958. 10.1093/hmg/ddi325 16126734

[B73] Liu Q. Zhou J. Daiger S. P. Farber D. B. Heckenlively J. R. Smith J. E.. (2002). Identification and subcellular localization of the RP1 protein in human and mouse photoreceptors. Invest. Ophthalmol. Vis. Sci. 43, 22–32. 11773008 PMC1963488

[B74] Liu Q. Zuo J. Pierce E. A. (2004). The retinitis pigmentosa 1 protein is a photoreceptor microtubule-associated protein. J. Neurosci. 24, 6427–6436. 10.1523/JNEUROSCI.1335-04.2004 15269252 PMC1904502

[B75] Liu Y. W. Curtis M. A. Gibbons H. M. Mee E. W. Bergin P. S. Teoh H. H.. (2008). Doublecortin expression in the normal and epileptic adult human brain. Eur. J. Neurosci. 28, 2254–2265. 10.1111/j.1460-9568.2008.06518.x 19046368

[B76] Maiato H. Sampaio P. Sunkel C. E. (2004). Microtubule-associated proteins and their essential roles during mitosis. Int. Rev. Cytol. 241, 53–153. 10.1016/S0074-7696(04)41002-X 15548419

[B77] Marin O. Valiente M. Ge X. Tsai L. H. (2010). Guiding neuronal cell migrations. Cold Spring Harb. Perspect. Biol. 2:a001834. 10.1101/cshperspect.a001834 20182622 PMC2828271

[B78] Marino C. Meng H. Mascheretti S. Rusconi M. Cope N. Giorda R.. (2012). DCDC2 genetic variants and susceptibility to developmental dyslexia. Psychiatr. Genet. 22, 25–30. 10.1097/YPG.0b013e32834acdb2 21881542 PMC3232293

[B79] Massinen S. Hokkanen M. E. Matsson H. Tammimies K. Tapia-Paez I. Dahlstrom-Heuser V.. (2011). Increased expression of the dyslexia candidate gene DCDC2 affects length and signaling of primary cilia in neurons. PLoS ONE 6:e20580. 10.1371/journal.pone.0020580 21698230 PMC3116825

[B80] Masui K. Mawatari S. Y. Suzuki S. O. Iwaki T. (2008). Evaluation of sensitivity and specificity of doublecortin immunostatining for the detection of infiltrating glioma cells. Brain Tumor Pathol. 25, 1–7. 10.1007/s10014-007-0225-1 18415660

[B81] Matsuo N. Kawamoto S. Matsubara K. Okubo K. (1998). Cloning and developmental expression of the murine homolog of doublecortin. Biochem. Biophys. Res. Commun. 252, 571–576. 10.1006/bbrc.1998.9698 9837748

[B82] Migaud M. Batailler M. Pillon D. Franceschini I. Malpaux B. (2011). Seasonal changes in cell proliferation in the adult sheep brain and pars tuberalis. J. Biol. Rhythms 26, 486–496. 10.1177/0748730411420062 22215607

[B83] Migaud M. Batailler M. Segura S. Duittoz A. Franceschini I. Pillon D. (2010). Emerging new sites for adult neurogenesis in the mammalian brain, a comparative study between the hypothalamus and the classical neurogenic zones. Eur. J. Neurosci. 32, 2042–2052. 10.1111/j.1460-9568.2010.07521.x 21143659

[B84] Miyata H. Chute D. J. Fink J. Villablanca P. Vinters H. V. (2004). Lissencephaly with agenesis of corpus callosum and rudimentary dysplastic cerebellum, a subtype of lissencephaly with cerebellar hypoplasia. Acta Neuropathol. 107, 69–81. 10.1007/s00401-003-0776-0 14566414

[B85] Mohr M. A. Sisk C. L. (2013). Pubertally born neurons and glia are functionally integrated into limbic and hypothalamic circuits of the male Syrian hamster. Proc. Natl. Acad. Sci. U.S.A. 110, 4792–4797. 10.1073/pnas.1219443110 23460698 PMC3607016

[B86] Moon H. M. Wynshaw-Boris A. (2013). Cytoskeleton in action, lissencephaly, a neuronal migration disorder. Wiley Interdiscip. Rev. Dev. Biol. 2, 229–245. 10.1002/wdev.67 23495356 PMC3593794

[B87] Moores C. A. Francis F. Perderiset M. Houdusse A. (2003). A double-take on MAPs. Nat. Struct. Biol. 10, 314–316. 10.1038/nsb0503-314 12714997

[B88] Moores C. A. Perderiset M. Francis F. Chelly J. Houdusse A. Milligan R. A. (2004). Mechanism of microtubule stabilization by doublecortin. Mol. Cell 14, 833–839. 10.1016/j.molcel.2004.06.009 15200960

[B89] Moores C. A. Perderiset M. Kappeler C. Kain S. Drummond D. Perkins S. J.. (2006). Distinct roles of doublecortin modulating the microtubule cytoskeleton. EMBO J. 25, 4448–4457. 10.1038/sj.emboj.7601335 16957770 PMC1590004

[B90] Nagayasu E. Hwang Y. C. Liu J. Murray J. M. Hu K. (2017). Loss of a doublecortin (DCX)-domain protein causes structural defects in a tubulin-based organelle of Toxoplasma gondii and impairs host-cell invasion. Mol. Biol. Cell 28, 411–428. 10.1091/mbc.E16-08-0587 27932494 PMC5341725

[B91] Nosten-Bertrand M. Kappeler C. Dinocourt C. Denis C. Germain J. Phan Dinh Tuy F. (2008). Epilepsy in Dcx knockout mice associated with discrete lamination defects and enhanced excitability in the hippocampus. PLoS ONE 3:e2473. 10.1371/journal.pone.0002473 18575605 PMC2429962

[B92] Ocbina P. J. Dizon M. L. Shin L. Szele F. G. (2006). Doublecortin is necessary for the migration of adult subventricular zone cells from neurospheres. Mol. Cell. Neurosci. 33, 126–135. 10.1016/j.mcn.2006.06.014 16931042

[B93] Ortensi B. Osti D. Pellegatta S. Pisati F. Brescia P. Fornasari L.. (2012). Rai is a new regulator of neural progenitor migration and glioblastoma invasion. Stem Cells 30, 817–832. 10.1002/stem.1056 22311806

[B94] Ortensi B. Setti M. Osti D. Pelicci G. (2013). Cancer stem cell contribution to glioblastoma invasiveness. Stem Cell Res. Ther. 4, 18. 10.1186/scrt166 23510696 PMC3706754

[B95] Ozer R. S. Halpain S. (2000). Phosphorylation-dependent localization of microtubule-associated protein MAP2c to the actin cytoskeleton. Mol. Biol. Cell 11, 3573–3587. 10.1091/mbc.11.10.3573 11029056 PMC15014

[B96] Pechnick R. N. Zonis S. Wawrowsky K. Pourmorady J. Chesnokova V. (2008). p21Cip1 restricts neuronal proliferation in the subgranular zone of the dentate gyrus of the hippocampus. Proc. Natl. Acad. Sci. U.S.A. 105, 1358–1363. 10.1073/pnas.0711030105 18172194 PMC2234143

[B97] Pierce A. A. Xu A. W. (2010). *De novo* neurogenesis in adult hypothalamus as a compensatory mechanism to regulate energy balance. J. Neurosci. 30, 723–730. 10.1523/JNEUROSCI.2479-09.2010 20071537 PMC3080014

[B98] Pramparo T. Youn Y. H. Yingling J. Hirotsune S. Wynshaw-Boris A. (2010). Novel embryonic neuronal migration and proliferation defects in Dcx mutant mice are exacerbated by Lis1 reduction. J. Neurosci. 30, 3002–3012. 10.1523/JNEUROSCI.4851-09.2010 20181597 PMC2861429

[B99] Reiner O. Coquelle F. M. Peter B. Levy T. Kaplan A. Sapir T.. (2006). The evolving doublecortin (DCX) superfamily. BMC Genomics 7:188. 10.1186/1471-2164-7-188 16869982 PMC1550402

[B100] Rich J. N. Hans C. Jones B. Iversen E. S. McLendon R. E. Rasheed B. K.. (2005). Gene expression profiling and genetic markers in glioblastoma survival. Cancer Res. 65, 4051–4058. 10.1158/0008-5472.CAN-04-3936 15899794

[B101] Robins S. C. Stewart I. McNay D. E. Taylor V. Giachino C. Goetz M.. (2013). α-Tanycytes of the adult hypothalamic third ventricle include distinct populations of FGF-responsive neural progenitors. Nat. Commun. 4:2049. 10.1038/ncomms3049 23804023

[B102] Rodriguez O. C. Schaefer A. W. Mandato C. A. Forscher P. Bement W. M. Waterman-Storer C. M. (2003). Conserved microtubule-actin interactions in cell movement and morphogenesis. Nat. Cell Biol. 5, 599–609. 10.1038/ncb0703-599 12833063

[B103] Salmon W. C. Adams M. C. Waterman-Storer C. M. (2002). Dual-wavelength fluorescent speckle microscopy reveals coupling of microtubule and actin movements in migrating cells. J. Cell Biol. 158, 31–37. 10.1083/jcb.200203022 12105180 PMC2173033

[B104] Sanchez-Farias N. Candal E. (2015). Doublecortin is widely expressed in the developing and adult retina of sharks. Exp. Eye Res. 134, 90–100. 10.1016/j.exer.2015.04.002 25849205

[B105] Santra M. Santra S. Buller B. Santra K. Nallani A. Chopp M. (2011). Effect of doublecortin on self-renewal and differentiation in brain tumor stem cells. Cancer Sci. 102, 1350–1357. 10.1111/j.1349-7006.2011.01952.x 21477071 PMC3116092

[B106] Santra M. Zheng X. Roberts C. Santra S. Lu M. Panda S.. (2010). Single doublecortin gene therapy significantly reduces glioma tumor volume. J. Neurosci. Res. 88, 304–314. 10.1002/jnr.22207 19681167 PMC2795007

[B107] Sapir T. Horesh D. Caspi M. Atlas R. Burgess H. A. Wolf S. G.. (2000). Doublecortin mutations cluster in evolutionarily conserved functional domains. Hum. Mol. Genet. 9, 703–712. 10.1093/hmg/9.5.703 10749977

[B108] Schaar B. T. Kinoshita K. McConnell S. K. (2004). Doublecortin microtubule affinity is regulated by a balance of kinase and phosphatase activity at the leading edge of migrating neurons. Neuron 41, 203–213. 10.1016/S0896-6273(03)00843-2 14741102

[B109] Schaefer A. W. Kabir N. Forscher P. (2002). Filopodia and actin arcs guide the assembly and transport of two populations of microtubules with unique dynamic parameters in neuronal growth cones. J. Cell Biol. 158, 139–152. 10.1083/jcb.200203038 12105186 PMC2173029

[B110] Sossey-Alaoui K. Hartung A. J. Guerrini R. Manchester D. K. Posar A. Puche-Mira A.. (1998). Human doublecortin (DCX) and the homologous gene in mouse encode a putative Ca^2+^-dependent signaling protein which is mutated in human X-linked neuronal migration defects. Hum. Mol. Genet. 7, 1327–1332. 10.1093/hmg/7.8.1327 9668176

[B111] Tanaka T. Serneo F. F. Tseng H. C. Kulkarni A. B. Tsai L. H. Gleeson J. G. (2004). Cdk5 phosphorylation of doublecortin ser297 regulates its effect on neuronal migration. Neuron 41, 215–227. 10.1016/S0896-6273(03)00852-3 14741103

[B112] Tint I. Jean D. Baas P. W. Black M. M. (2009). Doublecortin associates with microtubules preferentially in regions of the axon displaying actin-rich protrusive structures. J. Neurosci. 29, 10995–11010. 10.1523/JNEUROSCI.3399-09.2009 19726658 PMC2757270

[B113] Tozzini E. T. Baumgart M. Battistoni G. Cellerino A. (2012). Adult neurogenesis in the short-lived teleost Nothobranchius furzeri, localization of neurogenic niches, molecular characterization and effects of aging. Aging Cell 11, 241–251. 10.1111/j.1474-9726.2011.00781.x 22171971 PMC3437507

[B114] Tsukada M. Prokscha A. Oldekamp J. Eichele G. (2003). Identification of neurabin II as a novel doublecortin interacting protein. Mech. Dev. 120, 1033–1043. 10.1016/S0925-4773(03)00177-1 14550532

[B115] Tsukada M. Prokscha A. Ungewickell E. Eichele G. (2005). Doublecortin association with actin filaments is regulated by neurabin II. J. Biol. Chem. 280, 11361–11368. 10.1074/jbc.M405525200 15632197

[B116] Viprey V. F. Gregory W. M. Corrias M. V. Tchirkov A. Swerts K. Vicha A.. (2014). Neuroblastoma mRNAs predict outcome in children with stage 4 neuroblastoma, a European HR-NBL1/SIOPEN study. J. Clin. Oncol. 32, 1074–1083. 10.1200/JCO.2013.53.3604 24590653

[B117] von Bohlen und Halbach O. (2011). Immunohistological markers for proliferative events, gliogenesis, and neurogenesis within the adult hippocampus. Cell Tissue Res. 345, 1–19. 10.1007/s00441-011-1196-4 21647561

[B118] Wakabayashi T. Kosaka J. Mori T. Takamori Y. Yamada H. (2008). Doublecortin expression continues into adulthood in horizontal cells in the rat retina. Neurosci. Lett. 442, 249–252. 10.1016/j.neulet.2008.07.030 18647639

[B119] Wang C. Liu F. Liu Y. Y. Zhao C. H. You Y. Wang L.. (2011). Identification and characterization of neuroblasts in the subventricular zone and rostral migratory stream of the adult human brain. Cell Res. 21, 1534–1550. 10.1038/cr.2011.83 21577236 PMC3365638

[B120] Wang Y. Yin X. Rosen G. Gabel L. Guadiana S. M. Sarkisian M. R.. (2011). Dcdc2 knockout mice display exacerbated developmental disruptions following knockdown of doublecortin. Neuroscience 190, 398–408. 10.1016/j.neuroscience.2011.06.010 21689730 PMC3170724

[B121] Weimer J. M. Anton E. S. (2006). Doubling up on microtubule stabilizers, synergistic functions of doublecortin-like kinase and doublecortin in the developing cerebral cortex. Neuron 49, 3–4. 10.1016/j.neuron.2005.12.016 16387632

[B122] Xu Y. Tamamaki N. Noda T. Kimura K. Itokazu Y. Matsumoto N.. (2005). Neurogenesis in the ependymal layer of the adult rat 3rd ventricle. Exp. Neurol. 192, 251–264. 10.1016/j.expneurol.2004.12.021 15755543

[B123] Yamada M. Onodera M. Mizuno Y. Mochizuki H. (2004). Neurogenesis in olfactory bulb identified by retroviral labeling in normal and 1-methyl-4-phenyl-1,2,3,6-tetrahydropyridine-treated adult mice. Neuroscience 124, 173–181. 10.1016/j.neuroscience.2003.10.040 14960349

[B124] Yamamura T. Barker J. M. Balthazart J. Ball G. F. (2011). Androgens and estrogens synergistically regulate the expression of doublecortin and enhance neuronal recruitment in the song system of adult female canaries. J. Neurosci. 31, 9649–9657. 10.1523/JNEUROSCI.0088-11.2011 21715630 PMC3214644

[B125] Yanez Y. Hervas D. Grau E. Oltra S. Perez G. Palanca S.. (2016). TH and DCX mRNAs in peripheral blood and bone marrow predict outcome in metastatic neuroblastoma patients. J. Cancer Res. Clin. Oncol. 142, 573–580. 10.1007/s00432-015-2054-7 26498952 PMC11819078

[B126] Yap C. C. Digilio L. McMahon L. Roszkowska M. Bott C. J. Kruczek K.. (2016). Different Doublecortin (DCX) patient alleles show distinct phenotypes in cultured neurons, EVIDENCE FOR DIVERGENT LOSS-OF-FUNCTION AND “OFF-PATHWAY” CELLULAR MECHANISMS. J. Biol. Chem. 291, 26613–26626. 10.1074/jbc.M116.760777 27799303 PMC5207172

[B127] Zhao C. Deng W. Gage F. H. (2008). Mechanisms and functional implications of adult neurogenesis. Cell 132, 645–660. 10.1016/j.cell.2008.01.033 18295581

